# Solution-Phase Synthesis of KCl Nanocrystals Templated by PEO-PPO-PEO Triblock Copolymers Micelles

**DOI:** 10.3390/polym16070982

**Published:** 2024-04-03

**Authors:** Lingling Sun, Min Li, Fei Li, Fuchun Wang, Xiangfeng Liang, Qinghui Shou

**Affiliations:** 1 Qingdao Institute of Bioenergy and Bioprocess Technology (QIBEBT), Chinese Academy of Sciences (CAS), Qingdao 266101, China; sunll@qibebt.ac.cn (L.S.); limin@qibebt.ac.cn (M.L.); lifei@qibebt.ac.cn (F.L.); 2 Shandong Energy Institute, Qingdao 266101, China; 3 Qingdao New Energy Shandong Laboratory, Qingdao 266101, China; 4 School of Materials and Metallurgical Engineering, Guizhou Institute of Technology, Guiyang 550003, China; fuchunhelichun@126.com

**Keywords:** inorganic nanocrystal, hollow crystal, self-assembly, template, PEO-PPO-PEO, triblock copolymer

## Abstract

The current work introduces the synthesis of inorganic salt nano/micro-crystals during the reduction of hydrogen tetrachloroaurate(III) by Pluronic triblock copolymers (P123, PEO_20_–PPO_70_–PEO_20_). The morphologies and component were confirmed using an electron microscope with an electronic differential system (EDS), and the crystal structures were determined with X-ray diffraction (XRD). The morphologies highly depend on the concentrations of Pluronic and pH values. The mean size of the nanocrystal and hollow micro-crystal were controlled typically in the range of 32–150 nm (side length) and 1.4 μm, respectively. Different from the electrospray–ionization (EI) method, a model in which KCl forms a supersaturated solution in the micellar core of Pluronic is used to explain the formation process. This work provides the new insight that inorganic salt nanocrystals could be synthesized with the template of micelles in pure aqueous solutions.

## 1. Introduction

Nano/Micro-crystals have attracted wide attention due to their special physicochemical properties, and the widely developed fabrication techniques including “top-down” and “bottom-up” have paved the way for the practical use of nanocrystals in fields such as the analysis of environmental pollutants [1], interfacial catalysis [2,3], etc. Among them, KCl and NaCl inorganic salt nanocrystals exhibit promising features as standard materials because of their highly monodisperse structure [4]. They are also ideal candidates for the synthesis of hollow nanoparticles [5,6] and heteronanocrystals [7]. Recently, hollow NaCl crystals have also attracted attention considering their promising application, such as energy storage and drug delivery [8].

Compared with the fabrication of other inorganic nanocrystals such as transition metal oxide, NaYF_4_ upconversion nanocrystals, there are still challenges to synthesize KCl-type nanocrystals because they are highly water-soluble. Nanocrystals are mainly synthesized through the electrospray–ionization (EI) technique [9] and evaporation–sharp condensation technique [10]. In the widely adapted EI technique, dispersed and charged droplets with micro-scale were firstly created by the electrospray of solutions, and then the surface tensions of such droplets were reduced through the increasing electric field and ion strength. Finally, with the evaporation of water, nanocrystals were produced. The concentration of ions is important for the crystal formation. Quite recently, Fan et al. utilized this technique to synthesize NaCl hollow crystals [8]. As one typical “top-down” technique, the EI method often needs high temperature, high pressure, and high-cost equipment [9], and the obtained nanocrystals are easy to aggregate and have low monodispersity, which are not conducive to the practical use.

Epple et al. reported the synthesis of NaCl nanocrystals through the reaction between sodium diethyl malonate and phenacyl chloride. The released Cl^−^-resultant NaCl nanocrystals are covered with a thin layer of the organic materials [11]. Wang et al. prepared NaCl nanocubes with a mean diameter of 18 nm through the reaction of CF_3_COONa and HCl in the presence of oleic acid and oleylamine at above 300 °C [7]. Chen et al. developed a continued synthesis technique through the confluent of NaCl aqueous saturation solution and ethanol (anti-solvent) [5]. Wang et al. prepared NaCl nanocubes through dropping hot NaCl glycerol solution into ice-cooling monohydric alcohol [6]. These strategies utilize the sudden decreased solubility of NaCl in alcohol. In general, the “top-down” technique including EI often requires high temperature and high-price facilities [9], and the obtained nanocrystals by other methods are easy to aggregate.

The “bottom-up” technique has been widely used for the synthesis of nanocrystals due to its facile preparation conditions and low cost. Especially, the self-assembly method using surfactants and amphiphilic block copolymers as capping agents has been widely used for the synthesis of nanocrystals. Surfactants, which are composed of a hydrophilic head and a hydrophobic chain, can self-assemble into a rich variety of organized structures in solution, such as micelles, reverse micelles, vesicles, and lyotropic liquid crystals. Currently, reverse micelles have been adapted as nanostructured reactors for the synthesis of various inorganic nanostructures, such as semiconductors and oxides [12]. Both spherical nanoparticles and 1-D nanostructures are produced inside the aqueous microenvironment of the reverse micelles [13,14]. In comparison, the solution-phase synthesis of nanocrystals using normal micelles has rarely been reported. The hydrophobic core is segregated from the hydrophilic corona. Due to the high necessity of an aqueous environment, the crystallization is difficult to realize inside micelles. However, it is reported that there is still a certain amount of water in the hydrophobic core of micelles [15]. For example, poly(ethylene oxide)-poly(propylene oxide)-poly(ethylene oxide) (PEO-PPO-PEO, under the tradename Pluronic^®^ from BASF) triblock copolymers could self-assemble into close-packed micelles. The water-contained micellar core structure has been revealed through spectroscopic tools, such as Fourier-transform infrared spectroscopy (FT-IR) and small-angle neutron scattering analysis (SANS) [16,17]. Therefore, it is predicted that supersaturated solution in the micelles’ core could be formed, which facilitates the production of nanocrystals. Block copolymers have been widely used for the synthesis of metal nanoparticles. Research mainly focused on the shape and size control of them [18,19,20,21,22,23]. To our knowledge, there is no report on the synthesis of inorganic salt nanocrystals and hollow micro-crystals.

In this work, KCl and NaCl nanocrystals have been produced using potassium hydroxide (or sodium hydroxide) and hydrogen tetrachloroaurate(III) trihydrate as precursors and Pluronic triblock copolymer as a template, respectively. The structure of nanocrystals is confirmed with XRD and electron microscopy. The morphology highly depends on the concentrations of Pluronic and pH values. The medium size of nanocubes was controlled typically in the range of 32–150 nm (edge length) and 63 nm (diameter). TEM images show that KCl nanocrystals grow from the micellar core of Pluronic P123. A model in which KCl forms a supersaturated solution in the micellar core of Pluronic P123 was used to explain the formation mechanism. This article synthesized inorganic salt nanocrystals and hollow micro-crystals in the template of a block copolymer for the first time, which provides a new idea for the design of crystalline materials.

## 2. Material and Methods

### 2.1. Materials and Reagents

Sodium hydroxide and potassium hydroxide of analytical grade were purchased from Beijing Chemical Co. of China (Beijing, China). Hydrogen tetrachloroaurate(III) tetrahydrate (HAuCl_4_·3H_2_O) and Pluronic triblock copolymer P123 [EO_20_PO_70_EO_20_, Mw = 5750] were obtained from Sigma Aldrich (Shanghai, China) Trading Co., Ltd. of China. All reagents were used as received without further purification.

### 2.2. Synthesis of Inorganic Nanocrystals

Inorganic salt crystals were prepared by the soft template method with triblock copolymer P123 as the reducing and stabilizing agent. In a typical procedure, the pH value of the P123 stock solution was adjusted with a certain concentration of KOH or NaOH prior to use, and nanocrystals were prepared by mixing 0.4 mL of 2 × 10^−3^ mol/L HAuCl_4_·3H_2_O aqueous solution with 4 mL of Pluronic P123 solution with a range of concentration. After agitation by a vortex mixer for about 10 s, the samples were kept at 40 °C. The crystal growth tended to be stable overnight.

### 2.3. Characterization of Inorganic Nanocrystals

X-ray crystallography was obtained using a microdiffractometer (AXS D8-Advance, Bruker, Karlsruhe, Germany) with Cu-Kα radiation (λ = 1.5406 Å) at 0.02° per step under the operation condition of 30 kV and 40 mA. The structural analysis was conducted using the MDI Jade 5.0 program. A scanning electron microscope (SEM; S-4800, Hitachi, Tokyo, Japan) and transmission electron microscope (TEM, H-7650, Hitachi, Tokyo, Japan) were used for observation of morphology and composition.

## 3. Results and Discussion

The crystallographic structure and morphology were determined using X-ray diffraction (XRD) and an electron microscope (TEM and SEM), respectively. With different concentrations of Pluronic and pH values, different morphologies are formed.

### 3.1. Determination of Crystallographic Structure and Morphology

**Figure 1:**
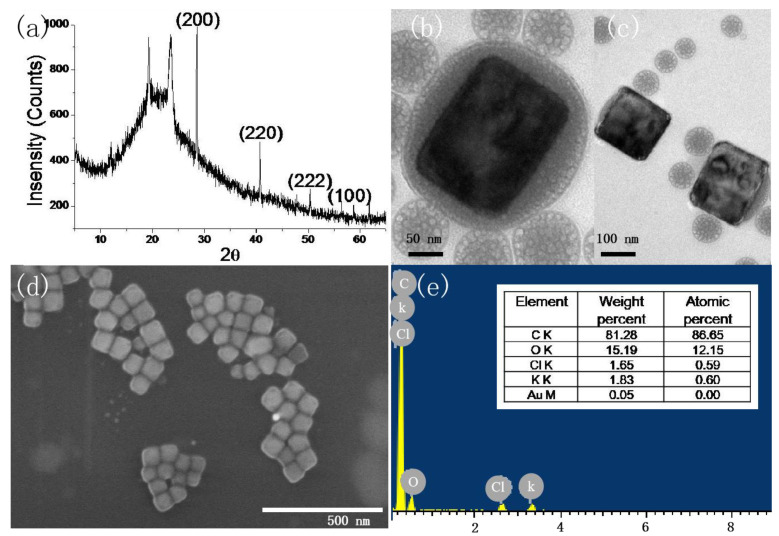
X-ray crystallographic (**a**), TEM (**b**,**c**), SEM (**d**), and energy-dispersive X-ray spectrometry (**e**) of KCl nanocubes (pH: 8.3, 50 g/L P123).

When the concentration of P123 is 50 g/L and the pH is 8.3, the morphology and component are firstly characterized. From the XRD pattern of the sample in Figure 1a, four peaks were observed at 28.350°, 40.510°, 50.160°, and 58.620°, corresponding to (200), (220), (222), and (100), respectively. KCl nanocrystal was indexed in a cubic unit cell of the space group Fm-3m [225] crystal orientation of the sample. In the pattern of neat PEO, there were two prominent diffraction peaks at 2θ = 19.3° and 23.4°, assigned to the (120) reflection and several overlapped reflections of the monoclinic crystal of PEO, respectively [24]. In the bare Si, only one peak was observed at 61.8°, corresponding to Si(024) [25]. The size of the nanocrystal is 81.2 nm, based on the Scherrer equation. As shown in Figure 1b–d, nanocubes with a mean side length of 66 nm were obtained. It is clearly observed that KCl nanocrystals are formed inside P123 micelles (Figure 1b,c). Energy-dispersive X-ray spectrometry (EDX) of nanocrystals shown in Figure 1e indicates that the composition is potassium and chlorides. The C and O elements are from the P123 matrix. Although a gold element is detected, its percent is very low compared with those of K and Cl. In the previous reported EI technique [9], nanocrystals with cubic shapes and 32 nm size are successfully grown from ultra-dilute NaCl solution (1 μg.mL^−1^) on a millisecond timescale. In the current work, the concentration of sodium ions is 44 μg.mL^−1^, and the nanocrystal size is on the same scale with that produced with the EI technique.

### 3.2. The Effect of the Concentration of Pluronic and pH

Pluronic assembles into rich morphologies, such as spherical micelles, rod-like micelles, vesicles, and lyotropic liquid crystals, which highly depend on the concentrations and temperatures. At 40 °C, Pluronic forms a gel above 300 g/L [26]. Therefore, a concentration of 5–250 g/L was studied. The concentration of chloroauric acid was kept constant at 0.2 mmol/L. Inorganic salt particles were synthesized by adjusting the pH of P123 with different concentrations of KOH (35 × 10^−3^ mM~600 × 10^−3^ mM). The morphology of KCL nanocrystals varying in Pluronic concentration and pH value is shown in Table 1. When the P123 concentration was 5 g/L, no inorganic salt particles were produced due to the slow reaction rate of chloroauric acid and the low K^+^ and CL^−^ concentrations; only gold nanoparticles were produced.

**Figure 2:**
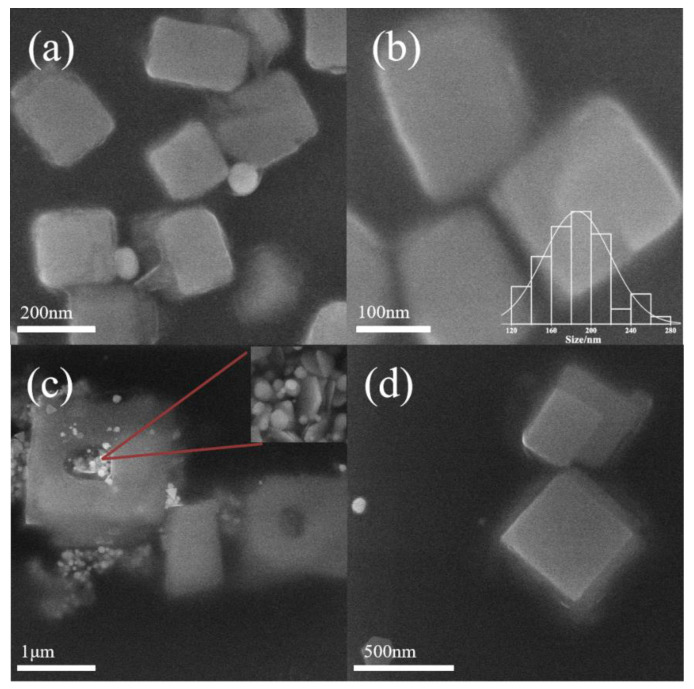
Morphologies of KCL nanocrystals at pH = 6.9 (**a**,**b**) and pH = 7.3 (**c**,**d**) with 10 g/L Pluronic P123. Notes: The bar chart represents the distribution of KCl crystallite sizes, and the red line indicates AuNPs. The appearance of hollow crystals caused the uneven particle size distribution, so the particle size distribution curve could not be provided.

**Figure 3:**
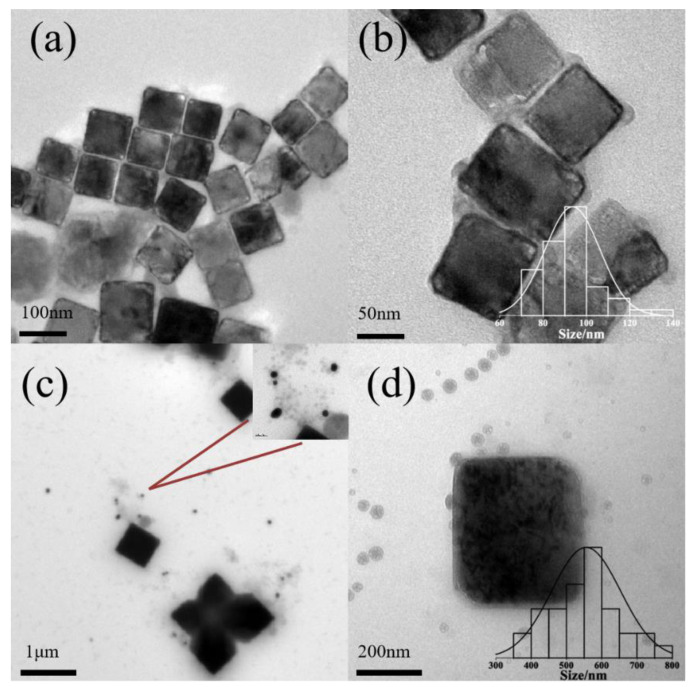
Morphologies of KCL nanocrystals at pH = 6.9 (**a**,**b**) and pH = 7.3 (**c**,**d**) with 50 g/L Pluronic P123. Notes: The bar chart represents the distribution of KCl crystallite sizes, and the red line indicates AuNPs.

**Figure 4:**
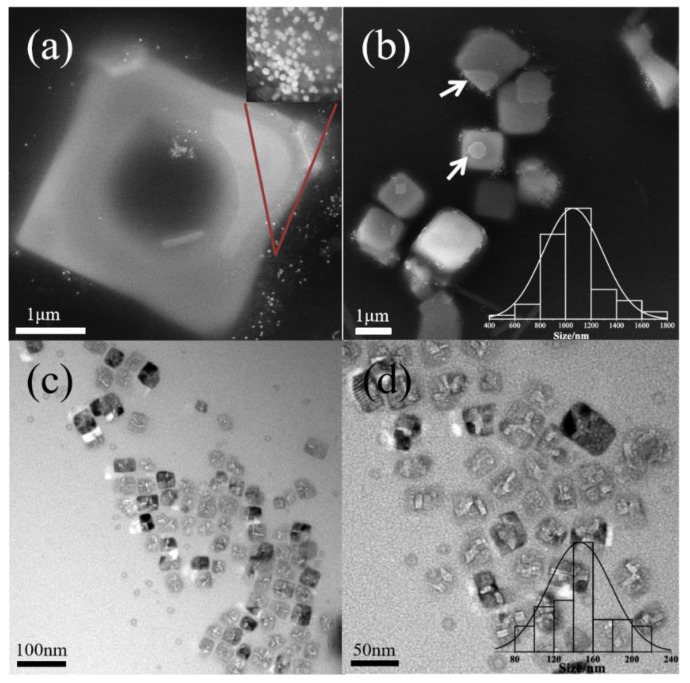
Morphologies of KCL nanocrystals at pH = 6.9 (SEM, (**a**,**b**)) and pH = 7.3 (TEM, (**c**,**d**)) with 100 g/L Pluronic P123. Notes: The bar chart represents the distribution of KCl crystallite sizes, and the red line indicates AuNPs.

When the P123 concentration was 10 g/L, inorganic salt nanoparticles were produced at pH 6.9, particle size up to 186 ± 33 nm (Figure 2a,b). Inorganic salt particles were produced within the micelle core. Meanwhile, as the pH increased to 7.9, hollow NaCl was produced (Figure 2c,d). Quite recently, hollow NaCl micro-crystals were synthesized through the EI technique [8]. The evaporation of the charged microdroplet drives single-point nucleation and outside-in growth. In this work, we confirmed for the first time such hollow structures could also be produced using block copolymer micelles. The inorganic salt particles reach the micron scale and produce hollow structures, which cannot be completed inside the micelle core. If nanoparticles are generated or added in block copolymer solutions (above the critical micelle solution), the single polymer chain adsorbs onto the surface of them [27]. Therefore, hollow crystals are assumed to be capped with P123 to remain stable in an aqueous solution. The hollow crystals are formed through many factors. Firstly, the micelles aggregate with increasing salt concentration [28]. The inorganic salt particles originally generated inside the micelle agglomerates further fuse with each other, but the concentration of inorganic salts inside the agglomerates after fusion is not enough to support its growth into a solid structure, leaving holes inside the inorganic salt particles. Secondly, the increase in salt concentration leads to water loss inside the micelles [29]. The concentration of K^+^ ions outside of the micelle is much higher than that inside of micelle, while Cl^−^ is in situ generated in the corona of the micelle during the reduction of tetrachloroaurate(III) by PEO. From Figure 3c, flower crystals are formed through the attachment of nanocrystals. This may also contribute to the micro-sized crystal. From Figure 4b, truncated microcubes and hollow microcubes with small holes are observed. Therefore, with the formation of nanocrystals that are capped with micelles or a single chain, the crystal growth continues to proceed on the surface of micelles. In this way, hollow crystals are formed.

With 50 g/L of P123 pH lower than pH 8.3, KCl nanocubes are also synthesized, as well as a small amount of rectangle-like nanocrystals. The sizes of nanocubes are 92 ± 12 nm (Figure 3a,b, pH 6.9) and 463 ± 128 nm (Figure 3c,d, pH 7.3), as shown in Figure 3. The morphology of micelles was also studied. The mean diameter of normal spherical micelles is less than 20 nm [30]. When the concentration of P123 is 50 g/L, the mean diameter of bare micelles is 41 ± 10.3 nm (Figure 3a,b, pH = 6.9) and 42 ± 6.6 nm (Figure 3c,d, pH = 7.3). Therefore, the size of micelles is increased with the formation of KCl nanocrystals. When the pH value is increased further, there is no observable formation of KCl nanocrystals. At pH 7.3, with the increase in salt concentration, micelles aggregate, and multiple inorganic salt particles aggregate to form flower-like crystals, which are the embryos of hollow crystals.

**Figure 5:**
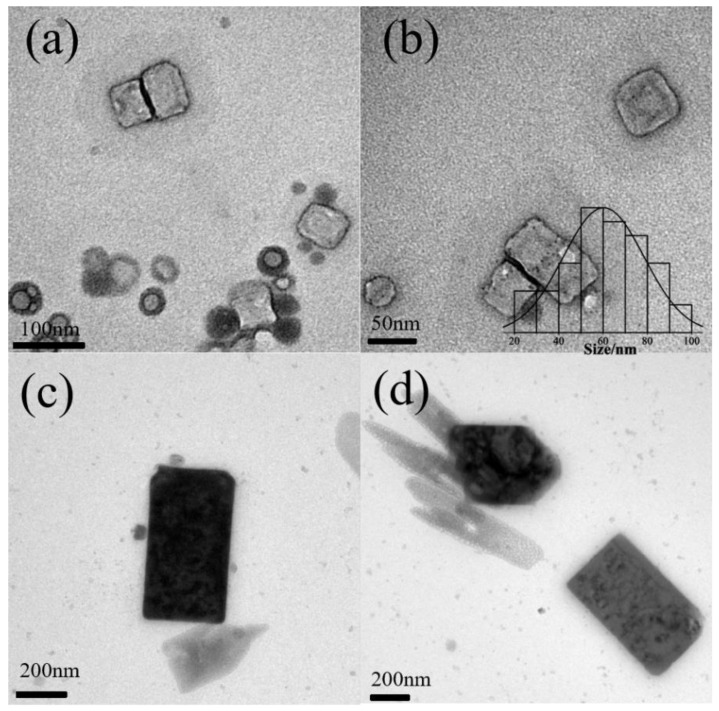
Morphologies of KCL nanocrystals at pH = 6.9 (**a**,**b**) and pH = 7.3 (**c**,**d**) with 200 g/L Pluronic P123. Notes: The bar chart represents the distribution of KCl crystallite sizes. Size distribution at pH 7.3 was not performed.

As the concentration of P123 increases to 100 g/L, the growth of nanocrystals is shown in Figure 4. Hollow KCl crystals is obtained when the pH is 6.9 (Figure 4a,b). The crystal size (1.4 μm) is much larger than that of micelles, which means that they cannot be directly formed through the template of micelles. When the pH is increased to 7.3, the size of KCl nanocrystals is 174 ± 37.6 nm (Figure 4c,d). With a further increase in salt concentration, the phenomenon of water loss inside the micelle globule is more serious, resulting in a decrease in the particle size of the formed inorganic salt nanoparticles, which can be completely stabilized by the micelle globule without aggregation. With the concentration of 200 g/L, the size of KCl nanocrystals is 57 ± 18 nm at pH 6.9 (Figure 5a,b) and 472 ± 176 nm at pH 7.3 (Figure 5c,d). When the P123 concentration approaches its gelation concentration, the micelle number density increases, leading to a denser micelle [28], slowing down the ion permeation rate and decreasing the ion concentration inside the micelle. So small-particle-size KCl nanocrystals were generated inside micelles at pH 6.9. And with the increase in pH, the salt concentration rose, and further dehydration occurred inside micelles, which could no longer meet the growth environment of inorganic salt nanocrystals, so KCl crystals grew outside micelles. In all cases, KCl nanocrystals are surrounded with thin polymer layers. In summary, KCl nanocubes can be produced when the concentration of P123 is in the range of 10–200 g/L and pH is lower than 8.3.

### 3.3. The Effect of the Type of Alkali Metal Ion

The synthesis of NaCl nanocrystals was also attempted using NaOH as precursors instead of KOH. The morphology diagram of NaCl nanocrystals is shown in Table S1. Comparing Table 1 and Table S1, the pH value and Pluronic concentration for the nanocrystals formation highly depend on the salt type. The size of NaCl nanocubes is similar with KCl (Figures S1 and S2). NaCl nanocubes formed in the more basic conditions and lower Pluronic concentration compared with that of KCl nanocubes. Pseudo ether structure is formed between cations and PEO or Pluronic with PEO segments [31]. Therefore, the association ability of different cations with Pluronic is important for the formations of nanocrystals. A detailed study on this point up to now is needed to explain the difference in KCl and NaCl nanocrystals formation.

The morphology of micelles is dependent on several factors. There is a hydrogen bond between the Pluronic polymer and water molecules. Increase in the pH value leads to the stretching of PEO chains and consequently results in the increase in the size of micelles [32].

Sakai et al. suggests that the formation of AuNPs from AuCl_4_ using PEO-PPO-PEO comprises three main steps [33]:

(1) Reduction of metal ions by block copolymers in solution and formation of gold clusters:

AuCl_4_^−^ + nPEO-PPO-PEO → Au^x^-(PEO-PPO-PEO)n → Au + 4Cl^−^ + 2H^+^ + oxidation products

(2) Adsorption of block copolymers on gold clusters and reduction of metal ions on the surfaces of those gold clusters:

Au_m_ + lPEO-PPO-PEO → lAu_m_-(PEO-PPO-PEO)

(3) Stabilization of gold particles by block copolymers

**Figure 6:**
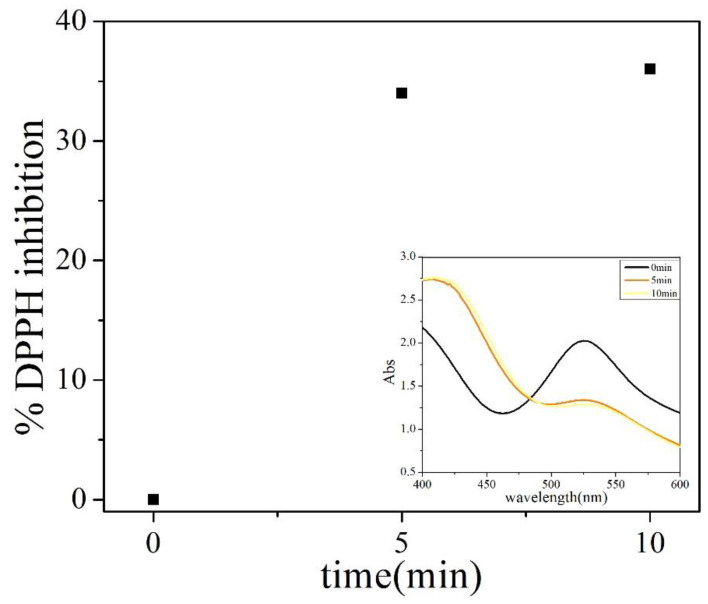
Time-dependent inhibition of DPPH: 0.4 mL HAuCl_4_ (1 mM) and DPPH (1 mM) solution were added into 4 mL 5 g/L P123. The spectra were measured from the beginning to 10 min. Note: The illustration shows the absorbance change of the reaction system after adding DPPH.

P123 acts as a reducing agent and stabilizer in the synthesis of AuNPs. Gomes et al. used cyclic voltammetry to test the redox potential of gold nanoparticles synthesized in the presence of the block copolymer Pluronic F127. F127 has an oxidation potential of +0.11 V, which is lower than the Au(III) reduction potential of +0.56 V, and therefore, F127 is able to reduce chloroaurate [34]. Since P123 has a similar molecular structure as F127, it is supposed to also have the capacity to reduce chloroaurate. Marina Sokolsky-Papkov demonstrated that the free radical mechanism dominates in the synthesis of gold nanoparticles from polymer composites. Through the characterization of gel permeation chromatography (GPC) and ^1^HNMR, the fragment of Pluronic after the reaction of the chloroaurate and Pluronic was detected [35]. In this work, the free radical scavenger DPPH was added during this reaction. As shown in Figure 6, after the addition of DPPH (0 min), a major absorbance was observed at 527 nm, which is a signal due to DPPH in its oxidized form. After 10 min, the adsorption clearly shows a decrease in the band at 527 nm and an increase in the band at 419 nm, which is the reduced form of DPPH (Figure 6 inset). DPPH reached an inhibition of 36% within 10 min (Figure 6), which clearly indicates the reduction of chloroaurate by P123 also generates free radicals.

Organic molecules possessing a hydroxyl group often have reducing capacity [36]. To study the role of the hydroxyl group of P123, carboxylic acid-ended P123 was synthesized. The successful modification was confirmed by the signal of the carboxylic group in ^1^HNMR (DMSO-d_6_ as solvent, Figure S3). When it was used for the same reaction, the nano-Au particles were not observed in the system show in Figure S4; only the change in colloidal morphology was observed. Based on all results, the formation of inorganic crystals and gold nanoparticles needs the involvement of both the polymer ether group and the hydroxyl group.

The effect of pH value on this reaction was investigated in our previous work [19] and by another researcher [37]. Synthesis of gold nanoparticles is typically determined by competition between nucleation (step 1) and growth (step 2 and 3) processes. Following the reaction kinetics of step 1 using UV–Vis spectroscopy, the reduction rate of gold ions is increased with increasing pH, and the size of gold nanoparticles is decreased. In the current work, the reduction rate of tetrachloroaurate influences the release of chloride ions, and the concentration of Cl^−^ is one of the key factors affecting KCl crystallization. At a low pH value and low Pluronic concentration, KCl crystallization could not happen due to the low release rate of Cl^−^ in the system. Correspondingly, the size of micelles did not change much. With the increase in pH value and Pluronic concentration, the size of micelles increased with the formation of both KCl nanocrystals and gold nanoparticles.

Although KCl nanocrystals are the by-product during the synthesis of gold nanoparticles, the yield of gold nanoparticles could be decreased through controlling the pH and polymer concentration. Moreover, there is a cheaper metal–chloride coordination compound, such as tetrachlorocopperic acid; therefore, this finding provides a new strategy to synthesize KCl and NaCl nano/micro-crystals, including hollow ones.

### 3.4. The Mechanism of Crystallization

The flexible PEO block dispersed in water and formed the corona of the micelle, and the hydrophobic PPO block formed the core of the micelle. It is reported that there is as much as a 20% volume fraction of water inside the Pluronic core at low temperatures (e.g., 35 °C) [17,38]. On the other hand, inorganic nanocrystals are synthesized with the electrospray–ionization (EI) technique [9]. First, dispersed and charged droplets with micro-scale were created by the electrospray of solutions; and then the surface tensions of such droplets were reduced with the electric field and ion strength; and finally, with the evaporation of the solvent, nanocrystals were produced. The concentration of ions is important for the crystal formation. Through the EI technique [11], nanocrystals with cubic shapes and 32 nm edges were successfully grown from ultra-dilute NaCl solution (1 μg.mL^−1^) on a millisecond timescale. In the current work, the concentration of sodium ions is 44 μg.mL^−1^, and the mean diameter is 32 nm, which is on the same scale with that produced with the ES technique. The concentration of chloride anions and potassium cations was calculated as shown in the supporting materials. The supersaturated solution of potassium chloride was formed.

The effect of inorganic salt on the micellization has been investigated widely, and it is reported that nanocrystals do not form when inorganic salt is added separately [39], indicating that it is necessary that the precursors of potassium and chloride are capped with polymer layers before they meet and form crystal. Especially, TEM images show that KCl nanocrystal grows from the micellar core of Pluronic^®^. Micelle turns into porous structure because of water evaporation during TEM specimen preparation. It is noteworthy that nanocrystals are grown from the micelle of Pluronic with the largest size. Therefore, it is concluded that KCl nanocrystals were synthesized inside Pluronic micelles, and it is predictable that there is a limit of the size of nanocrystals.

Based on the data above, the mechanisms that nanocrystals formed are proposed as below.

(1) Pseudo ether structures are formed between potassium cations and Pluronic, gold ions, and Pluronic micelles separately [31,40,41].
(2) Chloride anions are released with the deformation of hydrogen tetrachloroaurate(III) trihydrate.
(3) Potassium chloride nanocrystals are formed.

**Figure 7:**
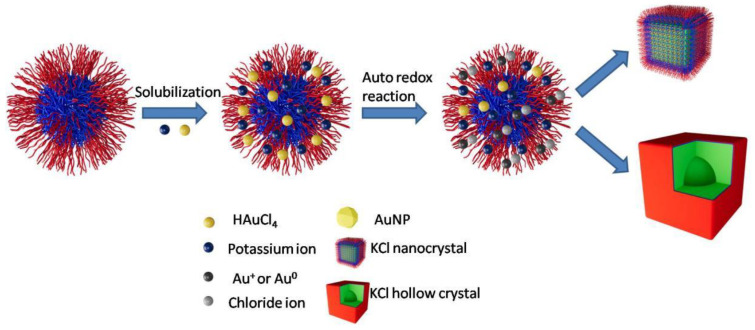
The formation mechanism of KCL nanocrystals and hollow micro-crystals.

Firstly, potassium cations and chloride anions are met with intermicellar exchange. Secondly, there is a small amount of water inside Pluronic micelles. With the amount of potassium and chloride increased, a supersaturated solution of potassium chloride is formed, and potassium chloride crystallizes from the solution. Finally, potassium and chloride are diffused into the micelles, and nanocrystals with a confined size are formed. The formation mechanism of KCL nanocrystals and hollow micro-crystals is shown in Figure 7.

## 4. Conclusions

A solution-phase method using PEO-PPO-PEO triblock copolymers has been developed for the synthesis of KCl and NaCl inorganic nanocrystals and hollow micro-crystals. The morphologies and sizes of nanocrystals highly depend on the PEO-PPO-PEO concentration and pH value. PEO-PPO-PEO micelles are performed as nanoreactors for the synthesis of nanocrystals. To the best of our knowledge, this is the first report on the synthesis of uniform KCl and NaCl nanocrystals using polymeric micelles. The current work has potential application in various fields, such as the templated synthesis of nanomaterials for the biomedical field or a heterogeneous catalyst for various organic reactions.

## Figures and Tables

**Table 1:** Morphologies of KCL nanocrystals with different P123 concentrations and pH values.

P123	pH 6.9	pH 7.3	pH 8.3	pH 9.5
Concentration				
5 g/L	-	-	-	-
10 g/L	Nanocubes	Nanocubes	-	-
50 g/L	Nanocubes	Nanocubes	Nanocubes	-
100 g/L	Nanocubes	Nanocubes	-	-
200 g/L	Nanocubes	Nanocubes	-	-
250 g/L	-	-	-	-

Note: “-” means there is no or few nanocrystals formed.

## Data Availability

Data are contained within the article.

## Supplementary Materials

The following supporting information can be downloaded at: https://www.mdpi.com/article/10.3390/polym16070982/s1, Figure S1: NaCl nanocubes at pH = 6.9 (a,b) with 1 w.t.% Pluronic P123. The scale bar is 1 μm, and 100 nm, respectively; Figure S2: NaCl nanocubes at pH = 8.3 (a,b), and pH = 9.5 (e,f) with 5 w.t.% Pluronic P123. The scale bar is 100 nm, 100 nm, 200 nm, and 100 nm, respectively; Table S1: The range of pH value and concentration of Pluronic on the NaCl nanocrystals formation.
