# Supplementary material for: Solution-Phase Synthesis of KCl Nanocrystals Templated by PEO-PPO-PEO Triblock Copolymers Micelles
Source: Polymers (Basel). 2024 Apr 3;16(7):982. doi: 10.3390/polym16070982 (PMC11013680; doi:10.3390/polym16070982)
Supplement: Supplementary file 1 [file polymers-16-00982-s001.zip › polymers-2911988-supplementary.pdf]

Solution phase synthesis of KCl nanocrystals templated by PEO-PPO-PEO triblock copolymers micelles

Lingling Sun [1,2,3], Min Li [1,2,3], Fei Li [1,2,3], Fuchun Wang [4], Xiangfeng Liang [1,2,3,*] and Qinghui Shou [1,2,3,*]

1 Qingdao Institute of Bioenergy and Bioprocess Technology (QIBEBT), Chinese Academy of Sciences (CAS), Qingdao 266101, China; sunll@qibebt.ac.cn (L.S.); limin@qibebt.ac.cn (M.L.); lifei@qibebt.ac.cn (F.L.)
2 Shandong Energy Institute, Qingdao 266101, China
3 Qingdao New Energy Shandong Laboratory, Qingdao 266101, China
4 School of Materials and Metallurgical Engineering, Guizhou Institute of Technology,
Guiyang 550003, China; fuchunhelichun@126.com
* Correspondence: liangxf@qibebt.ac.cn (X.L.); shouqh@qibebt.ac.cn (Q.S.)

Table of Contents

1. Calculation of concentration of chloride anions and potassium cations inside micelles
2. NaCl nanocrystals formation

Table S1 NaCl nanocrystals formation with pH value and Pluronic Concentration
Fig.S1 NaCl nanocubes with 10g/L Pluronic P123
Fig.S2 NaCl nanocubes with 50g/L Pluronic P123

1. The concentration of chloride anions and potassium cations was calculated as shown in the supporting materials. The supersaturated solution of potassium chloride is form ed.

KCl solution in the micelle:

(1). Water content on the single micellar core

Taken 10 w.t.% P123 as an example

P123 concentration: $21.6*10^{-6}$ mol/mL

Number concentration: $13*10^{18}$/mL

Aggregation number: 99

Concentration of micelles: $26*10^{16}$/mL

Mass of single micelle: $0.77*10^{-18}$ g

The water content in corona is taken as 5% (Ref. 1). The water mass in single corona: $0.38*10^{-19}$ g

(2). KCl concentration in corona

The dissociation of $AuCl^{-}_{4}$ is carried out inside of micelle. Nearly 100% of $AuCl^{-}_{4}$ is dissociated according to ICP-MS measurement.

The concentration of $AuCl^{-}_{4}$ is $1*10^{-2}$ mol/L. After mixing with P123 (The volume of P123 and $AuCl^{-}_{4}$ is 10:1.), the concentration decreases to $0.9*10^{-3}$ mol/L. The concentration of Cl^{-} is four times of that of $AuCl^{-}_{4}$.

Presuming that all of Cl^{-} is inside of micelle, and associated with K^{+} after dissociation.

The percentage of micelles loading with KCl is 6.8% calculated with TEM microscope.

The number of Cl^{-} inside every micelle is 123.8.

The concentration of Cl^{-} inside every micelle is: 5.3 mol/L (density: 1 g/mL).

The molecular weight of KCl is 74.55 g/moL.

The concentration of KCl in every micelle is: 398.6 g/L.

Considering the saturated concentration of KCl (401 g/L), crystallization is feasible under this condition.

Table S1. The range of pH value and concentration of Pluronic on the NaCl nanocrystals formation.

**P123 concentration**	**pH6.9**	**pH7.3**	**pH8.3**	**pH9.5**
10 g/L	Nanocubes	-	-	-
50 g/L	Nanocubes	Nanocubes	Nanocubes	Nanocubes
100 g/L	Nanocubes	Nanocubes	-	-
200 g/L	-	-	-	-
250 g/L	-	-	-	-

Note: “-” means there is no nanocrystals formed.

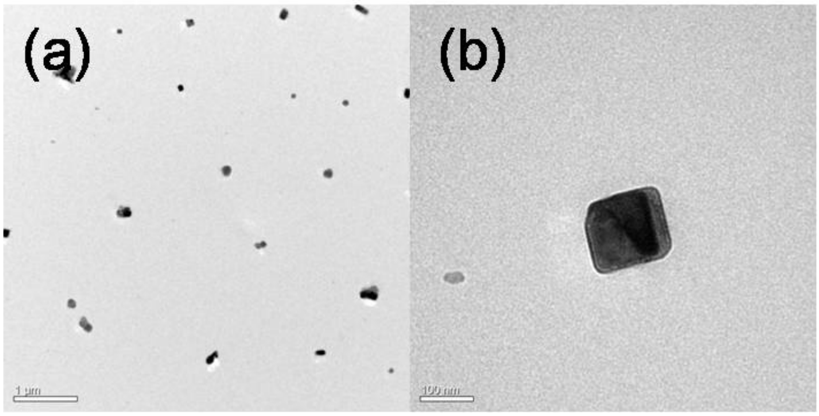

Figure S1. NaCl nanocubes at pH=6.9 (a, b) with 1 w.t.% Pluronic P123. The scale bar is 1 μm, and 100 nm, respectively.

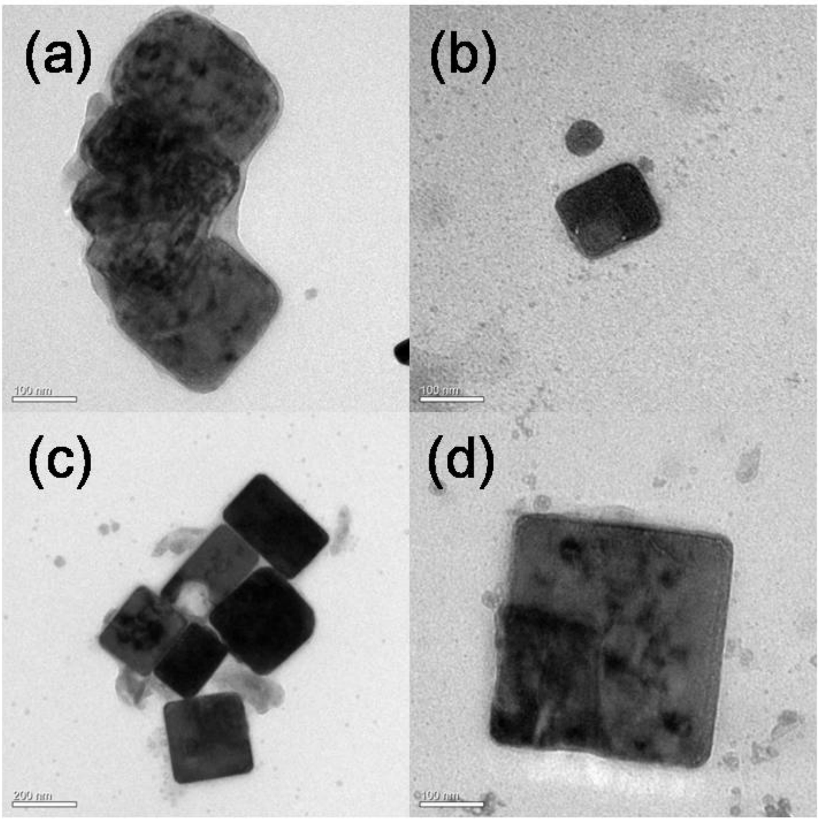

Figure S2. NaCl nanocubes at pH=8.3 (a, b), and pH=9.5 (c, d) with 5 w.t.% Pluronic P123. The scale bar is 100 nm, 100 nm, 200 nm, and 100 nm, respectively.

Reference

1. Nakashima, K.; Takeuchi, K. Water content in micelles of poly(ethylene oxide)-poly(propylene oxide)-poly(ethylene oxide) triblock copolymers in aqueous solutions as studied by fluorescence spectroscopy. *Appl. Spectrosc.* **2001**, *55*, 1237-1244, doi:Doi 10.1366/0003702011953252.
